# Healthcare-associated infection management in 62 ICUs for patients with congenital heart disease in China: a survey study

**DOI:** 10.1097/JS9.0000000000001138

**Published:** 2024-02-06

**Authors:** Xiaofeng Wang, Shuo Li, Da Huo, Shilin Wang, Wenlong Wang, Hongxia He, Qian Zhang, Jiantao Li, Xu Wang

**Affiliations:** a Department of Pediatric Intensive Care Unit, National Center for Cardiovascular Disease and Fuwai Hospital, Chinese Academy of Medical Sciences, Peking Union Medical College; b Department of Infection Control, National Center for Cardiovascular Disease and Fuwai Hospital, Chinese Academy of Medical Sciences, Peking Union Medical College; c Department of Infection Control, Peking University First Hospital; d Institute for Infectious Disease and Endemic Disease Control, Beijing Center for Disease Prevention and Control, Beijing, China

**Keywords:** antimicrobial stewardship, congenital heart disease, healthcare-associated infection, infection prevention and control, intensive care unit

## Abstract

**Objectives:**

All patients with congenital heart disease (CHD) receive postoperative management in ICUs. Infection prevention and control (IPC) has a significant impact on prognosis. This study provides a preliminary understanding of the fundamental aspects of IPC in ICUs following CHD surgery in China.

**Methods:**

From September to October 2023, we initiated a survey on healthcare-associated infection (HAI) management in hospitals that perform CHD surgeries independently. The questionnaires were jointly completed by the ICU physicians and IPC personnel. Duplicate or unqualified questionnaires were excluded from the study. The contents of our questionnaires covered hospital and ICU capacity, performance of the infection control department, HAI surveillance, implementation of IPC measures, and antimicrobial stewardship (AMS). Qualified questionnaires were compared according to the volume of annual CHD surgeries performed in different ICUs. Group 1 was defined as volume more than 300 cases and group 2 was defined as volume less than or equal to 300 cases.

**Results:**

Sixty-two of the 118 questionnaires were completed, with a response rate of 53%. The CHD surgical volume in 2022 of the 62 hospitals was 36342, accounting for 52% of the annual CHD surgical volume (69 672) across the country. The postoperative infection rates obtained from the 15 ICUs varied from 1.3 to 15%, with a median rate of 4.5%. A total of 16 ICUs provided data on drug-resistant bacteria, *Klebsiella pneumoniae* exhibiting the highest frequency. More than 95% of ICUs have established complete HAI management systems. Information-based HAI surveillance was conducted in 89% of ICUs. Approximately 67% of ICUs stopped prophylactic antibiotics within 48 hours after surgery. In complex cases, carbapenems were administered empirically in 89% of ICUs. Group 1 had an advantage over group 2 in preventing multi-drug-resistant organisms (all instruments should be used alone 100% vs. 86%, *P*=0.047; cleaning and disinfection of environmental surfaces, 100% vs. 81%, *P*=0.035; antibiotic consumption control 85% vs. 61%, *P*=0.044) and in preventing surgical site infections (perioperative blood glucose monitoring, 88% vs. 67%, *P*=0.048). However, Group 1 did not perform well in preventing catheter-related bloodstream infection (delayed catheter removal due to convenience of laboratory tests, 31% vs. 6%, *P*=0.021) and catheter-associated urinary tract infection (delayed catheter removal due to muscle relaxant administration, 88% vs. 58%, *P*=0.022).

**Conclusions:**

A relatively complete HAI management system has been established throughout the country in ICUs for CHD patients. Information-based surveillance of HAI needs to be promoted, and actions should be taken to facilitate the implementation of IPC measures and AMS bundles. Training and feedback are critical for implementing IPC measures.

## Introduction

Highlights

- A nationwide survey on infection prevention and control.
- Covers half of Chinese patients after congenital cardiac surgery.
- Clearly points out the progress and shortcomings.

Congenital heart disease (CHD) is the most common congenital malformation in China, with a comprehensive incidence of 1%^1^. Approximately 70 000 patients with CHD undergo surgical treatment annually^2^, all patients require treatment in the ICU after surgery. Many factors affect postoperative recovery, and the level of infection prevention and control (IPC) is very important^3,4^. To achieve the best performance of IPC, it is necessary to build an IPC system, enhance the monitoring function, and ensure that all IPC measures are in place, which is a complex systematic profession.

China, a large developing country, exhibits notable variations in its medical conditions across diverse regions. The past 3 years of the COVID-19 epidemic have witnessed tremendous developments in healthcare-associated infection (HAI) control in China^5^ and other developing countries^6,7^. However, room for improvement may remain in the implementation of IPC measures. This study provides a preliminary understanding of the fundamental aspects of IPC in ICUs following CHD surgery in China. It also serves as a foundation for future enhancements of HAI after CHD surgery.

## Methods

### Study design

The questionnaires were jointly completed by the ICU physicians and IPC personnel. Duplicate or unqualified questionnaires were excluded from the study. The contents of our questionnaires covered hospital and ICU capacity, performance of the infection control department, HAI surveillance, implementation of IPC measures, and antimicrobial stewardship (AMS). The questionnaire consisted of single-choice, multiple-choice, and open-response questions (typical answers are described in the results). Additionally, instructions were provided to the questionnaires to elucidate any pertinent professional terminology.

### Participants

From September to October 2023, we sent the electronic version of the survey via WeChat or E-mail to 118 hospitals that could perform CHD surgeries independently. The survey did not include invitations to hospitals in Hong Kong, Macao, and Taiwan. Duplicate or unqualified questionnaires were excluded.

### Procedures

**Figure 1:**
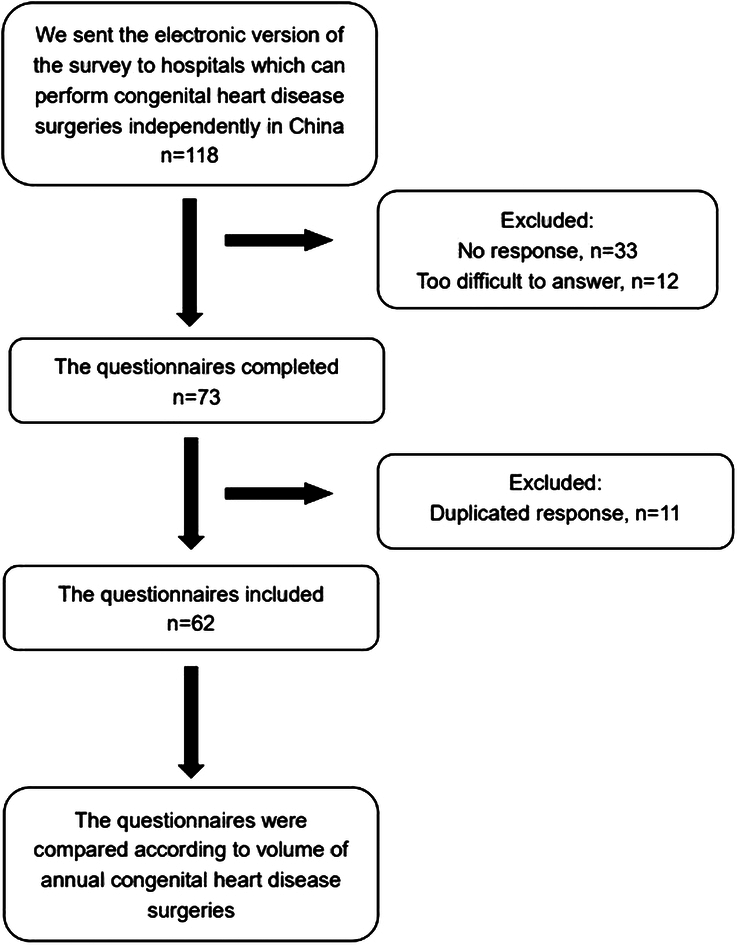
The flowchart of the steps in the methodology.

We introduced the purpose of the questionnaires, and because the questions were highly professional, it was recommended that the questionnaires be completed in collaboration with ICU doctors and IPC Personnel. After the questionnaires were collected, the data were summarized in a database and analyzed. After the questionnaires were collected, their quality was checked to clarify and verify the answers. For problems such as logic errors, missing items, and ambiguities, we contacted the person who filled out the questionnaires to verify the content. Qualified questionnaires were compared according to the volume of annual CHD surgeries performed in different ICUs^8^. Group 1 was defined as volume more than 300 cases and group 2 was defined as volume less than or equal to 300 cases. Each item of the questionnaire was compared to explore the differences in IPC levels between the two ICU groups. The steps of the methodology are shown in Fig. 1 as a flowchart.

### Statistical analysis

Statistical analysis was performed using SPSS 25.0 software. The Shapiro–Wilk test was used to assess the conformity of the continuous variables to the normal distribution. Normal distribution was expressed as the mean plus or minus the standard deviation, while non-normal distribution was expressed as the median accompanied by the interquartile range. Categorical variables are displayed in a numerical form, accompanied by their respective percentages. Categorical variables were analyzed using the χ^2^ test, correction for continuity, or Fisher’s exact test. Statistical significance was set than 0.05.

## Results

Sixty-two of 118 questionnaires were completed with a response rate of 53%. The CHD surgical volume in 2022 of 62 hospitals included in this study was 36 342, accounting for 52% of the annual CHD surgical volume (69 672) across the country. No response to the questionnaires and the high difficulty of the questionnaires were the main reasons for failing to complete, and duplicated questionnaires were the main reasons for exclusion.

The postoperative infection rates obtained from the 15 ICUs varied from 1.3 to 15%, with a median rate of 4.5%. A total of 16 ICUs provided data on 21 common multi-drug-resistant organisms (MDRO), including *Klebsiella pneumoniae* ×9, *methicillin-resistant Staphylococcus aureus* ×5, *Acinetobacter baumannii* ×4, *Escherichia coli* ×1, and *Pseudomonas aeruginosa* ×1. The results of the basic characteristics are shown in Table 1, the results of the performance of the infection control department are shown in Table 2, the results of HAI surveillance are shown in Table 3, the results of implementation of IPC measures are shown in Table 4, and the results of AMS are shown in Table 5.

**Table 1:** Basic characters of hospitals and ICUs.

Items	Number, *n* (%)
Type of hospital	
Teaching hospital	54 (87)
Non-teaching hospital	8 (13)
Per capita GDP	
Above the national level	36 (58)
Below the national level	26 (42)
Type of ICU	
Cardiac ICU	45 (73)
General ICU	17 (27)
No. ICU beds	
1–10	17 (27)
11–20	20 (32)
21–30	15 (25)
>30	10 (16)
Number of CHD surgeries in 2022	
<300	36 (58)
300–999	17 (27)
1000–2000	4 (7)
>2000	5 (8)
Age of CHD patients	
Only children (age ＜18 years)	13 (21)
Only adult (age ≥18 years)	12 (19)
Children and adult	37 (60)

CHD, congenital heart disease: GDP, gross domestic product.

**Table 2:** Performance of infection control department.

Items	Total number, *n* (%)	Group 1 number, *n* (%)	Group 2 number, *n* (%)	*P*
Is there an independent hospital IPC department				1.0
Yes	62 (100)	26 (100)	36 (100)	
No	0	0	0	
Regular report on antimicrobial resistance in hospital				1.0
Yes	61 (98)	26 (100)	35 (97)	
No	1 (2)	0	1 (3)	
Consultation of infectious disease specialists for infection management				1.0
Yes	61 (98)	26 (100)	35 (97)	
No	1 (2)	0	1 (3)	
Regular IPC meeting in hospital				1.0
Yes	55 (89)	23 (88)	32 (89)	
No	7 (11)	3 (12)	4 (11)	
Part-time IPC personnel in ICU				1.0
Yes	62 (100)	26 (100)	36 (100)	
No	0	0	0	
The profession of part-time IPC personnel in ICU				0.904
Nurse only	3 (5)	2 (8)	3 (8)	
Doctor only	16 (26)	6 (23)	10 (28)	
Doctor and Nurse	43 (69)	18 (69)	23 (64)	
Is there regular AMS meeting in hospital				0.35
Yes	51 (82)	20 (77)	31 (86)	
No	11 (18)	6 (3)	5 (14)	

AMS, antimicrobial stewardship; IPC, infection prevention and control.

**Table 3:** Healthcare-associated infection surveillance.

Items	Total number, *n* (%)	Group 1 number, n (%)	Group 2 number, *n* (%)	*P*
How does the hospital monitor the infection				
Prospective active surveillance (active investigation to obtain the occurrence of infection in hospitalized patients)	50 (81)	23 (88)	27 (75)	0.318
Retrospective active surveillance (retrospective investigation of infection by reviewing medical records of discharged patients)	41 (66)	19 (73)	22 (61)	0.326
Prospective passive surveillance (relying on clinicians to report infections in hospitalized patients)	43 (69)	17 (65)	26 (72)	0.564
Retrospective passive surveillance (relying on clinicians to report nosocomial infections at or after hospital discharge)	26 (42)	13 (50)	13 (36)	0.274
No surveillance	2 (3)	0	2 (6)	0.505
The function of healthcare-associated infection monitoring system				
Identify and alert to infection case	49 (79)	22 (85)	27 (75)	0.547
Identify and alert to infection outbreak	34 (55)	17 (65)	17 (47)	0.156
Report infection case	59 (95)	24 (92)	35 (97)	0.772
Statistical analysis	39 (63)	19 (73)	20 (56)	0.159
No informational surveillance system	7 (11)	1 (4)	6 (17)	0.243
Does the ICU monitor for catheter-related bloodstream infection				1.0
Yes	62 (100)	26 (100)	36 (100)	
No	0	0	0	
Does the ICU monitor for catheter-associated urinary tract infection				0.132
Yes	58 (94)	26 (100)	32 (89)	
No	4 (6)	0	4 (11)	
Does the ICU monitor for ventilator-associated pneumonia				1.0
Yes	61 (98)	26 (100)	35 (97)	
No	1 (2)	0	1 (3)	
Does the ICU monitor for surgical site infection				0.505
Yes	60 (97)	26 (100)	34 (94)	
No	2 (3)	0	2 (6)	
What kinds of multi-drug-resistant organism are monitored				
*Methicillin-resistant Staphylococcus aureus*	56 (90)	24 (92)	32 (89)	0.65
*Carbapenem-resistant Enterobacteriaceae*	52 (84)	21 (81)	31 (86)	0.573
*Carbapenem-resistant Acinetobacter baumannii*	53 (85)	22 (85)	31 (86)	0.869
*Carbapenem-resistant Pseudomonas aeruginosa*	49 (79)	22 (85)	27 (75)	0.547
*Vancomycin-resistant Enterococci*	42 (68)	18 (69)	24 (67)	0.831
*Extended Spectrum Beta-Lactamases*	42 (68)	18 (69)	24 (67)	0.831
No surveillance	2 (3)	1 (4)	1 (3)	0.815
The microbiological detection techniques				
Culture	62 (100)	26 (100)	36 (100)	1.0
Antibiotic sensitivity test	58 (94)	25 (96)	33 (92)	0.853
Polymerase chain reaction	42 (68)	19 (73)	23 (64)	0.445
High-throughput sequencing/ Next-generation sequencing	37 (60)	15 (58)	22 (61)	0.787
Microbial homology analysis	9 (15)	3 (12)	6 (17)	0.841

**Table 4:** Implementation of infection prevention and control measures.

Items	Total number, *n* (%)	Group 1 number, *n* (%)	Group 2 number, *n* (%)	*P*
Hand hygiene monitoring methods				
Field investigation	60 (97)	26 (100)	34 (94)	0.505
Video investigation	17 (27)	6 (23)	11 (31)	0.515
Estimating the consumption of hand hygiene products	27 (44)	12 (46)	15 (42)	0.725
No surveillance	2 (3)	1 (4)	1 (3)	1.0
Method of air disinfection				
Natural ventilation	29 (47)	17 (65)	12 (33)	0.013^*^
Mechanical ventilation	26 (42)	12 (46)	14 (39)	0.567
Ultraviolet disinfection	51 (82)	23 (88)	28 (78)	0.453
Laminar flow ventilation	50 (81)	23 (88)	27 (75)	0.318
Air disinfection machine	37 (60)	17 (65)	20 (56)	0.436
What is the frequency of cleaning and disinfection of environmental surfaces in ICU?				0.56
Once a day	21 (34)	9 (35)	12 (33)	
2 times per day	23 (37)	8 (31)	15 (42)	
3 times per day or more	17 (27)	8 (31)	9 (25)	
Only after the patient was discharged	1 (2)	1 (4)	1 (3)	
How to clean/disinfect/sterilize reusable devices in ICU?				0.187
Sent to central sterile supply department	50 (81)	21 (81)	29 (81)	
Only surgical instruments were sent to central sterile supply department	2 (3)	2 (8)	0	
Self-cleaning, send to the central sterile supply department	10 (16)	3 (12)	7 (19)	
Self-reuse	0	0	0	
What is the frequency of environmental hygiene surveillance in ICU?				0.08
Once a month	43 (69)	21 (81)	22 (61)	
Once three months	9 (15)	4 (15)	5 (14)	
Irregular surveillance	10 (16)	1 (4)	9 (25)	
The prevention and control measures for multi-drug-resistant organism	47 (76)	20 (77)	27 (75)	0.861
Active screening	56 (90)	24 (92)	32 (89)	0.65
Single room isolation	60 (97)	25 (96)	35 (97)	1.0
Bed side isolation	60 (97)	26 (100)	34 (94)	0.505
Isolation sign	60 (97)	26 (100)	34 (94)	0.505
Hand hygiene	57 (92)	26 (100)	31 (86)	0.047^*^
All instruments should be used alone	56 (90)	25 (96)	31 (86)	0.376
Wear gloves when caring for patients	55 (89)	25 (96)	30 (83)	0.243
Wear protective clothing in risk of contamination	55 (89)	26 (100)	29 (81)	0.035^*^
Cleaning and disinfection of environmental surfaces Antibiotic consumption in defined daily dose	44 (71)	22 (85)	22 (61)	0.044^*^
How does hospitals/ICUs prevent surgical site infections?				
Bathing one day before surgery	34 (55)	15 (58)	19 (53)	0.701
Prepare skin by shearing	47 (76)	18 (69)	19 (53)	0.304
Regulation of prophylactic antibiotics	56 (90)	24 (92)	32 (89)	0.65
Prolonged course of prophylactic antibiotics	28 (45)	13 (50)	15 (42)	0.515
Perioperative heat preservation	45 (73)	18 (69)	27 (75)	0.615
Perioperative blood glucose monitoring	47 (76)	23 (88)	24 (67)	0.048^*^
Chlorhexidine for skin disinfection (>2 months)				0.787
Yes	37 (60)	15 (58)	22 (61)	
No	25 (40)	11 (42)	14 (39)	
Ultrasound-guided central venous catheter insertion				1.0
Yes	58 (94)	24 (92)	34 (94)	
No	4 (6)	2 (8)	2 (6)	
Maximal sterile barriers during central venous catheter insertion				0.573
Yes	57 (92)	25 (96)	32 (89)	
No	5 (8)	1 (4)	4 (11)	
Replace the central line regularly?				0.794
Yes	51 (82)	21 (81)	30 (83)	
No	11 (18)	5 (19)	6 (17)	
What may lead to delayed removal of central venous catheter?				
Vasoactive agent infusion is required	50 (81)	22 (85)	28 (78)	0.729
Intravenous nutrient therapy is required	55 (89)	21 (81)	31 (81)	0.723
Central venous pressure monitoring	39 (63)	17 (65)	22 (61)	0.731
Complex cardiac malformations/surgical procedures	36 (50)	16 (62)	20 (56)	0.638
Convenient for laboratory tests	10 (16)	8 (31)	2 (6)	0.021^*^
Facilitate emergency medicine administration	54 (87)	25 (96)	29 (81)	0.154
Other	2 (3)	0	2 (6)	0.505
Urine culture diagnostic Stewardship when urine character changed				0.788
Direct urine culture	33 (53)	13 (50)	20 (56)	
Conditional urine culture based on urine white blood cell	26 (42)	12 (46)	14 (39)	
Never urine culture	1 (2)	0	1 (3)	
Other	2 (3)	1 (4)	1 (3)	
Replace the urinary catheter regularly?				0.505
Yes	60 (97)	26 (100)	34 (94)	
No	2 (3)	0	2 (6)	
What may lead to delayed removal of urinary catheter?				
Muscle relaxant administration	44 (71)	23 (88)	21 (58)	0.022^*^
Mechanical ventilation	42 (68)	21 (81)	21 (58)	0.062
Noninvasive ventilator assistance	17 (27)	9 (35)	8 (22)	0.28
Complex cardiac malformations/surgical procedures	37 (60)	15 (58)	22 (61)	0.787
Massive dose of diuretics	26 (42)	13 (50)	13 (36)	0.274
Worry about urinary retention	38 (61)	15 (59)	23 (64)	0.621
It facilitates measuring and monitoring fluid balance	40 (65)	19 (73)	21 (58)	0.231
Renal replacement therapy	35 (56)	17 (65)	18 (50)	0.228
Other	4 (6)	1 (4)	3 (8)	0.853
Is early extubation/fast-track surgery implemented in ICUs?				0.911
Yes	54 (87)	22 (85)	32 (89)	
No	8 (13)	4 (15)	4 (11)	
Are high-flow nasal cannula/noninvasive ventilator available?				1.0
Yes	61 (98)	26 (100)	35 (97)	
No	1 (2)	0	1 (3)	
Early tracheotomy for patients cannot wean from mechanical ventilation?				0.326
Yes	41 (66)	19 (73)	22 (61)	
no	21 (34)	7 (27)	14 (39)	
Replace the ventilator circuit regularly?				1.0
Yes	60 (97)	25 (96)	35 (97)	
no	2 (3)	1 (4)	1 (3)	

* P<0.05.

**Table 5:** Antimicrobial stewardship.

Items	Total number, *n* (%)	Group 1 number, *n* (%)	Group 2 number, *n* (%)	*P*
Postoperative prophylactic antibiotics				
Anti-cocci antibiotics	33 (53)	15 (58)	18 (50)	0.549
Anti-bacilli antibiotics	18 (29)	7 (27)	11 (31)	0.758
Broad-spectrum antibiotics	26 (42)	11 (42)	15 (42)	0.96
No prophylactic antibiotics	2 (3)	1 (4)	1 (3)	1.0
The course of prophylactic use of antibiotics				0.346
24 h	13 (21)	6 (23)	7 (19)	
48 h	28 (45)	10 (38)	18 (50)	
72 h	19 (31)	8 (31)	11 (31)	
No prophylactic antibiotics	2 (3)	2 (8)	0 (0)	
Therapeutic strategies for bacterial colonization				0.287
Leave it untreated	35 (56)	11 (42)	24 (67)	
Local use of antibiotics	5 (8)	3 (12)	2 (6)	
Systemic use of antibiotics	17 (27)	9 (35)	8 (22)	
Other	5 (8)	3 (12)	2 (6)	
Always take relevant cultures before commencing antibiotics				0.772
Yes	59 (95)	24 (92)	35 (97)	
No	3 (5)	2 (8)	1 (3)	
Empiric use of carbapenem antibiotics for critical ill patients				1.0
Yes	55 (89)	23 (88)	32 (89)	
No	7 (11)	3 (11)	4 (11)	
The decision of antifungal agent is based on				0.586
Clinical judgment	11 (18)	6 (23)	5 (14)	
Results of the G/GM test	24 (39)	11 (42)	13 (36)	
Results of fungal culture	25 (40)	8 (31)	17 (47)	
Other	2 (3)	1 (4)	1 (3)	
The first-line antifungal agent				0.226
Fluconazole	35 (57)	12 (46)	23 (64)	
Caspofungin	12 (19)	7 (27)	5 (14)	
Voriconazole	12 (19)	5 (19)	7 (19)	
Amphotericin B/ liposome	1 (2)	0	1 (3)	
Other	2 (3)	2 (8)	0	

All the surveyed hospitals were tertiary hospitals, half of the hospitals performed less than 300 CHD surgeries, and nine hospitals performed more than 1000 CHD surgeries in 2022. The results of this survey show that all hospitals and ICUs were appointed with full-time and part-time IPC personnel. The survey revealed that HAI surveillance has been established in hospitals and ICUs, and nearly 90% of hospitals and ICUs have built up information-based surveillance. In the implementation of IPC measures, hand hygiene monitoring is highly achieved in ICUs, and meticulous attention is paid to the cleaning and disinfection of environmental surfaces. There is a potential risk associated with the supply of reusable medical devices. There is significant heterogeneity in IPC measures for surgical site infection. The phenomenon of overuse of antibiotics exists in ~30% of ICUs based on the results of AMS.

When comparing the questionnaires between the two groups, group 1 had an advantage over group 2 in preventing MDRO (all instruments should be used alone 100% vs. 86%, *P*=0.047; cleaning and disinfection of environmental surfaces, 100% vs. 81%, *P*=0.035; antibiotic consumption control 85% vs. 61%, *P*=0.044) and in preventing surgical site infections (perioperative blood glucose monitoring, 88% vs. 67%, *P*=0.048). However, Group 1 did not perform well in preventing catheter-related bloodstream infection (delayed catheter removal due to convenience of laboratory tests, 31% vs. 6%, *P*=0.021) and catheter-associated urinary tract infection (delayed catheter removal due to muscle relaxant administration, 88% vs. 58%, *P*=0.022). There was also a statistically significant difference in the method of air disinfection (natural ventilation 65% vs. 33%, *P*=0.013) between the two groups.

## Discussion

### The basic characters of hospitals and ICUs

Postoperative management is very important for the surgical treatment of CHD patients, in which all the surveyed hospitals belong to tertiary hospitals, 87% of them are teaching hospitals, and 81% can complete paediatric cardiac procedures. After the operation, 71% of the patients received treatment in the cardiac ICUs.

In 2022, more than half of the hospitals performed fewer than 300 CHD surgeries, and only 9 hospitals performed more than 1000 CHD surgeries. Moreover, more than half of the CHD surgical centres are located in 11 economically developed Provinces/Autonomous Regions/Centrally Administered Municipalities (defined as the per capita gross domestic product exceeding the national level of 81 000 CNY in 2022), whereas the remaining 20 Provinces/Autonomous Regions/Centrally Administered Municipalities have less than 50% of CHD surgical centres. Based on these fundamental observations, it can be inferred that China, a large developing country, exhibits notable variations in medical conditions across diverse regions.

In this study, data on the postoperative nosocomial infection rate were collected from 15 ICUs, which ranged from 1.3 to 15%, with a median of 4.5%. These findings are similar to the data reported in the Society of Thoracic Surgeons Congenital Heart Surgery database (0.9–9.5% at the hospital level and 3.7% at the patient level) in 2013^9^, indicating that the efforts of HAI in China have made progress. Nevertheless, only 25% of the surveyed ICUs provided infection rate data, primarily because this is the first time that such a survey was conducted in China, and to improve the response rate, the infection rate is an optional item. Furthermore, the prevailing MDRO observed in this study are gram-negative bacilli, which is consistent with the current literature on MDRO^10^.

### The performance of infection control department

Following the COVID-19 outbreak, the importance of IPC and HAI has received unprecedented attention. The Chinese government has issued public documents to assist with the construction of IPC management systems in hospitals. The results of this survey show that all hospitals and ICUs were appointed with full-time and part-time IPC personnel. The cooperation of full-time and part-time IPC personnel also makes the IPC team play a better role in difficult infection cases^11^. In addition, ~70% of ICUs have built IPC teams with the cooperation of doctors and nurses. More than 80% of hospital administrators attach great importance to IPC and hold regular hospital-level meetings to discuss the problems encountered in HAI and AMS.

### The HAI surveillance

HAI surveillance forms a pillar of the IPC system. The survey revealed that HAI surveillance has been established in hospitals and ICUs. More than 90% of ICUs perform well in both comprehensive and targeted surveillance (encompassing device-associated infection, surgical site infection, and MDRO). However, the mode of IPC personnel actively carrying out HAI surveillance has not been fully established, and the proportion of clinical staff actively reporting nosocomial infection cases is unsatisfactory. A considerable proportion of hospitals persist in conducting retrospective surveillance examinations of the medical records of discharged patients. This approach fails to detect nosocomial infections or to promptly identify suspected outbreaks of nosocomial infections.

European and North American nations have established information-based network systems to improve the quality and efficiency of HAI surveillance and IPC management. China is also working hard on this project with fruitful results^12^. In this survey, nearly 90% of hospitals and ICUs built up information-based surveillance, although functions that reflect the depth of surveillance, such as identifying or alerting suspected outbreaks of nosocomial infection and statistical analysis, need to be further refined.

### The implementation of IPC measures

Owing to the important role of hand hygiene in IPC^13^, hand hygiene monitoring is highly achieved in ICUs. However, the mode of direct monitoring is dominant, while the proportion of indirect monitoring methods, such as estimation of the consumption of hand hygiene products, is low, reflecting the probably overestimated objectivity of hand hygiene compliance.

According to the survey results, meticulous attention is given to the cleaning and disinfection of environmental surfaces; however, some ICUs do not conduct regular monitoring, which may increase the risk of nosocomial infections.

The survey also found a potential risk in the supply process of reusable medical devices because some ICUs failed to follow the standard centralized disinfection and sterilization processes, although the proportion was very small (3%). We also inquire that this is because the ICUs are equipped with advanced medical devices, but the central sterile supply department is not equipped with the corresponding sterilization capacity; hence, the ICUs purchase sterilization equipment by themselves.

Because MDRO can increase hospital stay and medical costs and even increase mortality^14^, it is necessary to focus on prevention and control. Commonly used IPC measures include targeted surveillance, interruption of transmission (hand hygiene, isolation, and environmental hygiene), and AMS^15^. Additionally, it has been observed that 75% of the ICUs have carried out active screening for MDRO.

There is significant heterogeneity in IPC measures for surgical site infection. Only half of the hospitals practice preoperative bodywash; nearly half of the ICUs extend the course of antimicrobial therapy to prevent surgical site infection, and 40% of ICUs do not use chlorhexidine for skin disinfection in patients aged older than 2 months^16^. These findings indicate that significant effort is required for further enhancement.

There was no problem with the operation technique in the IPC measure for catheter-related bloodstream infection, such as ultrasound-guided insertion and maximal sterile barriers. The ICU doctors provided possible reasons for the delayed removal of the central venous catheter. The three most common causes are vasoactive agent infusion, intravenous nutrient therapy, and facilitation of emergency medicine administration. These reasons align with the characteristics of delayed recovery following complex CHD surgery and prolonged need for a central venous catheter. Comprehensive supervision measures should be enhanced to avoid catheter-related bloodstream infection^17^.

In the IPC measure for catheter-associated urinary tract infection, only 40% of ICUs perform urine culture based on urine white blood cell count, which may lead to overdiagnosis of urinary tract infection^18^. The ICU doctors also provided possible reasons for the delayed removal of the urinary catheter. The two most common causes are muscle relaxant administration and mechanical ventilation. These reasons also align with the characteristics of complex CHD surgery; for the same reason, comprehensive supervision measures should be enhanced^17^.

In the IPC measure for ventilator-associated pneumonia, 87% of ICUs have implemented early extubation/fast-track surgery, 98% of ICUs can provide high-flow nasal cannula/noninvasive ventilator for suitable patients, and 66% of ICUs choose early tracheotomy for patients who cannot wean from mechanical ventilation^19^. The results of this part of the questionnaire were the most satisfactory.

### The AMS

In recent years, antimicrobial stewardship in Chinese hospitals has achieved remarkable results^20^. The prophylactic use of antibiotics after CHD surgery is recommended, but the course should be limited to within 48 h^21^. The phenomenon of overuse of antibiotics exists in ~30% of ICUs and should be considered. For bacterial colonization, nearly 30% of ICUs chose systemic application of antibiotics, which also demonstrates the phenomenon of excessive antibiotic prescription. Empiric use of carbapenem antibiotics was common in our survey (89% of ICUs), which is the result of critically ill conditions after complex CHD surgeries. It is reasonable to take cultures before commencing antibiotics and prepare to scale down or cease after the culture results for critically ill patients^22^.

Regarding antifungal agents, 79% of the ICUs refer to culture or laboratory test results. Half of the ICUs selected fluconazole as first-line medication. However, nearly 40% of ICUs select caspofungin or voriconazole as the first-line medication because of the inevitable immune function impairment and poor prognosis of fungal infections in patients following cardiopulmonary bypass^23^.

### The differences between the two groups

In terms of air disinfection, there is a difference in the natural ventilation method. There were no differences in the other air disinfection methods (such as ultraviolet disinfection and laminar flow ventilation). This phenomenon may be attributed to the layout of the ICU buildings. Considering that this does not have an impact on the outcome of IPC management, this item is not a point of future improvement. More importantly, Group 1 has more advantages than Group 2 in preventing MDRO (all instruments should be used alone; cleaning and disinfection of environmental surfaces; antibiotic consumption control) and in preventing surgical site infections (perioperative blood glucose monitoring). These deficiencies are closely related to the prognosis of patients after cardiac surgery and are key points for improvement in these less-experienced ICUs in the future. In addition to IPC management, less-experienced ICUs should pay more attention to other factors, such as malnutrition^24^. Malnutrition is an additional risk factor for mortality and morbidity in ICU patients, particularly in those with CHD^25^. Patients with CHD often have increased metabolic demands and may experience challenges in maintaining adequate nutrition owing to feeding difficulties or gastrointestinal issues. Malnutrition can compromise immune function, impair wound healing, and exacerbate strain on the cardiovascular system in individuals with CHD. The intricate relationship between malnutrition and CHD further amplifies the complexity of care in ICU settings. Adequate nutritional support is crucial to address these vulnerabilities and requires a multidisciplinary approach involving dietitians, paediatric cardiologists, and critical care teams. Patients with conditions such as sepsis, acute respiratory distress syndrome, or multiorgan failure often have compromised physiological functions, placing them at a higher risk of adverse outcomes^26^.

However, group 1 did not perform well in preventing catheter-related bloodstream infection (delayed catheter removal due to convenience of laboratory tests) and catheter-associated urinary tract infection (delayed catheter removal due to muscle relaxant administration). Although this phenomenon may be related to the higher complexity of cardiac malformation, it is still recommended that these experienced ICUs pay attention to the rapid recovery of postoperative patients because the above two infections are important indicators to judge the IPC level and are related to the prognosis of patients.

### Study limitations

Fewer ICUs are available with data on the postoperative infection rate and MDRO. A possible reason cannot be ruled out is that some ICUs with high infection rates refuse to provide their data. To extract HAI surveillance data in accordance with international standards and implement uniform IPC measures^27^, we suggest that the next step would be to establish a national CHD surgery-related HAI surveillance network.

## Conclusions

A relatively complete HAI management system has been established throughout the country in ICUs for CHD patients. Information-based surveillance of HAI needs to be promoted, and actions should be taken to facilitate the implementation of IPC measures and AMS bundles. Training and feedback are critical for implementing IPC measures.

## Ethical statement

The study was approved by the Ethics Committee of Fuwai Hospital (approval number 2022-1732. Because it did not involve the inclusion of patients, the requirement for informed consent was waived.

## Consent

Our article is based on a series of questionnaire compiled in response to doctors' responses and therefore does not involve patient informed consent. Because it did not involve the inclusion of patients, the informed consent was waved.

## Sources of funding

This work was supported by the Clinical Research Foundation of National Health Commission of the People’s Republic of China (grant number 2022-GSP-GG-32).

## Author contribution

X.W., S.L., D.H. contributed to design and improvement of the questionnaire, overall quality control of the questionnaire. X.W. contributed to article writing and article revision. S.W. contributed to distribution of questionnaires, recovery of questionnaires, statistical analysis of the data. W.W. contributed to screen the target population of the questionnaire. H.H. contributed to answer the questions of the respondents. Q.Z., J.L. contributed to data entry, data sorting and data discussion. X.W. contributed to the overall project design.

## Conflicts of interest disclosure

The authors declare that the research was conducted in the absence of any commercial or financial relationships that could be construed as potential conflicts of interest.

## Research registration unique identifying number (UIN)

Our article did not address the participation of human samples and was therefore not prospectively registered on these sites.

## Guarantor

Dr Xu Wang is the people who accept full responsibility for the work and/or the conduct of the study, had access to the data, and controlled the decision to publish.

## Data sharing statement

All the data collected for the study, including survey data and related documents such as study protocol and statistical analysis results, will be made available to others. The data that support the findings of this study are available from the corresponding author, [Dr Xiaofeng Wang], upon reasonable request.

## Provenance and peer review

Our paper was not invited.
